# Theoretical Analysis of the Dynamic Properties of a 2-2 Cement-Based Piezoelectric Dual-Layer Stacked Sensor under Impact Load

**DOI:** 10.3390/s17051019

**Published:** 2017-05-04

**Authors:** Taotao Zhang, Yangchao Liao, Keping Zhang, Jun Chen

**Affiliations:** School of Transportation Science and Engineering, Beihang University, Beijing 100191, China; zhangtt@buaa.edu.cn (T.Z.); liaoyc@buaa.edu.cn (Y.L.); zy1613308@buaa.edu.cn (K.Z.)

**Keywords:** cement-based piezoelectric sensor, impact load, dynamic properties, theoretical solutions, numerical analysis, variable separation method, Duhamel integral

## Abstract

Cement-based piezoelectric materials are widely used due to the fact that compared with common smart materials, they overcome the defects of structure-incompatibility and frequency inconsistency with a concrete structure. However, the present understanding of the mechanical behavior of cement-based piezoelectric smart materials under impact load is still limited. The dynamic characteristics under impact load are of importance, for example, for studying the anti-collision properties of engineering structures and aircraft takeoff-landing safety. Therefore, in this paper, an analytical model was proposed to investigate the dynamic properties of a 2-2 cement-based piezoelectric dual-layer stacked sensor under impact load based on the piezoelectric effect. Theoretical solutions are obtained by utilizing the variable separation and Duhamel integral method. To simulate the impact load and verify the theory, three types of loads, including atransient step load, isosceles triangle load and haversine wave load, are considered and the comparisons between the theoretical results, Li’s results and numerical results are presented by using the control variate method and good agreement is found. Furthermore, the influences of several parameters were discussed and other conclusions about this sensor are also given. This should prove very helpful for the design and optimization of the 2-2 cement-based piezoelectric dual-layer stacked sensor in engineering.

## 1. Introduction

Cement-based piezoelectric sensors, a new kind of functional structure developed in recent decades, are fabricated from a cement matrix and piezoelectric ceramic phase in different volume fractions and using various mixing rules [1,2]. Cement-based piezoelectric composites have very sensitive transduction properties as well as good compatibility with the most popular construction materials (such as cement and concrete) used in civil engineering. They have received much research attention in recent years and have great potential application as a novel kind of electromechanical sensor material in structural health monitoring, which makes it crucial to study the overall properties of cement-based piezoelectric composites for sensor design, practical engineering application and optimization [3,4,5].

Most of the studies have focused on the preparation of cement-based piezoelectric sensors and determining their relevant parameters by experimental methods. By using a cut-filling process, Huang et al. prepared 2-2 cement-based piezoelectric composites [6,7]. In their paper, the effects of ceramic volume fraction and water-cement ratio on the properties of the composites were studied. The results indicated that the piezoelectric strain constant increases rapidly with increasing volume fraction of ceramic, while the water-cement ratio has little influence on the piezoelectric properties of the composite. Li et al. prepared 2-2 cement-based piezoelectric composites and two kinds of properties of the actuator and sensor—converse piezoelectric effect and piezoelectric effect—were introduced [8,9]. Using embedded 1-3 cement-based piezoelectric sensors, Qin et al. have prepared plain concrete and engineered cement composite beams [10]. Active and passive detection of beams’ damage evolution could be performed with these sensors. Cheng et al. have prepared a 1-3 cement-based piezoelectric ceramic composite [11]. In their essay, the influences of temperature, aspect ratios of piezoelectric ceramic rods and piezoelectric ceramic volume fraction on the dielectric and piezoelectric properties of the composites were studied. By using the dice-and-fill technique, Xu et al. prepared 2-2 cement-based piezoelectric composites [12]. In their paper, the effects of cement matrix and composite thickness on the acoustic and electrical properties of the composites were researched. They have also fabricated 2-2 cement/polymer based piezoelectric composites with inorganic fillers and investigated the effects of filler content and composite thickness on the properties of the composites [13]. Gong et al. have fabricated cement-based piezoelectric composites containing carbon nanotubes and found that the addition of nanotubes significantly enhances the piezoelectric properties of the composites [14]. Yang et al. have researched the sensitivity of the electromechanical admittance and the structural mechanical impedance to damages in a concrete structure [15]. Xing et al. have researched the influences of the impedance spectra of cement-based piezoelectric composites and studied the pore structure and its effects on the properties of composites [16,17]. Chaipanich et al. have investigated the dielectric properties of 2-2 connectivity lead magnesium niobate-lead titanate cement composites [18,19]. They have also researched the influences of 1-year ageing on the piezoelectric coefficient of the composite after poling. Chaipanich et al. have further prepared 0–3 piezoelectric lead zirconate titanate ceramic-cement composites, then the ferroelectric hysteresis behavior and piezoelectric force microscope characterization of the composites were studied [20]. Li et al. have developed a new type of cement-based piezoelectric sensor to monitor traffic flows in the field of transportation [21].

Meanwhile, studies on the dynamic characteristics of cement-based piezoelectric sensors are still relatively limited. Until now, only a few theoretical studies have dealt with the analysis of cement-based piezoelectric composites. Han et al. obtained theoretical solutions for four kinds of cement-based piezoelectric composites under external load. The influences of the polarization direction and material parameters on the theoretical solutions were studied and the relationship between the blocking force and the applied voltage of the actuators was obtained [22]. By using the displacement method, Zhang et al. have studied the dynamic characteristics of 2-2 cement-based piezoelectric sensors under the influence of external sinusoidal electrical potential and external sinusoidal pressure [23]. Zhang et al. have also studied the dynamic properties of piezoelectric structures under impact load and the theoretical solutions of the mechanical and electrical fields of the piezoelectric structure were obtained with the standing and traveling wave methods [24]. Because a dynamic load can cause serious damage to the composite structure, it is meaningful to study the theoretical dynamic characteristics of cement-based piezoelectric composites, especially under impact load.

In a setting of the anti-collision properties of engineering structures and aircraft takeoff-landing safety, this paper focuses on the properties of a 2-2 cement-based piezoelectric dual-layer stacked sensor under impact load. The basic equations are given in Section 2 based on the theory of piezo-elasticity. Next in Section 3, by combining these equations and boundary conditions, theoretical solutions of 2-2 cement-based piezoelectric dual-layer stacked sensor are obtained by utilizing the variable separation method and Duhamel integral. In Section 4, comparisons between the theoretical results, Li’s results [25] and numerical results are presented and discussed using the control variate method and good agreement is found. During the numerical calculation, the transient step load, the transient isosceles triangle load and transient haversine wave load are used to simulate impact loads, respectively. The influences of the thickness of piezoelectric layer and the parameters of the piezoelectric material are discussed, thus verifying the validity of the theoretical solutions. Finally, a summary and conclusions are presented. This study should be very helpful for the design and optimization of 2-2 cement-based piezoelectric dual-layer stacked sensors in engineering.

## 2. Basic Equations

**Figure 1:**
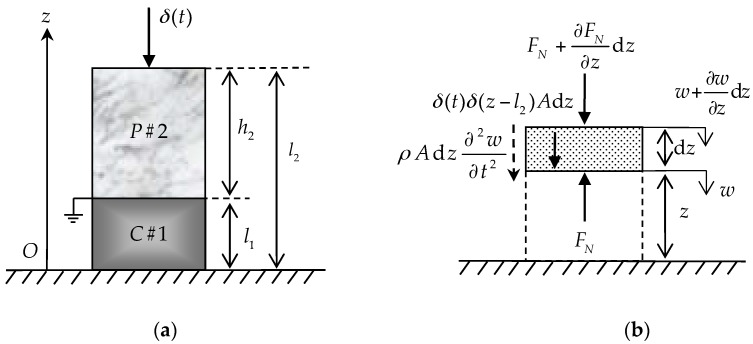
Theoretical analysis diagram: (**a**) Schematic of 2-2 cement-based piezoelectric dual-layer stacked sensor; (**b**) Force analysis of the element in the longitudinal vibration of sensor.

Figure 1a is a schematic of a 2-2 cement-based piezoelectric dual-layer stacked sensor with one fixed end and the other free. The bottom and top layers of the sensor, denoted as C#1 (thickness $l_{1}$ ) and P#2 (thickness $h_{2}$ ), are the cement and piezoelectric layer, respectively. The free end of the sensor is subjected to an impact load δ(t) . The symbols D , E , ε and σ denote the electric displacement, electric field, strain and stress, respectively, with reference to the Cartesian coordinate system. Linear elastic material is assumed for the material of the cement and piezoelectric layer. Figure 1b is a force analysis diagram of the element in the longitudinal vibration of sensor.

According to Figure 1b and Newton’s second law, we can get the equilibrium condition of the sensor longitudinal arbitrary unit body:

(1) $$\rho Adz\frac{\partial^{2}w}{\partial t^{2}}=\frac{\partial F_{N}}{\partial z}dz+\delta (t)\delta (z-l_{2})Adz$$

Here A , ρ and w are, respectively, the cross section, density and displacement of the specific cement and piezoelectric layer. $\delta (t)\delta (z-l_{2})$ suggests that the sensor is subjected to the impact load at the free end. The expressions for δ(t) and $\delta (z-l_{2})$ can be written as:

(2) $$\left\{ \begin{array}{l} \delta (t)=\left\{ \begin{array}{l} \infty ,\;t=0\\ 0,\;t\neq 0 \end{array}\right.\\ \delta (z-l_{2})=\left\{ \begin{array}{l} \infty ,\;z=l_{2}\\ 0,\;z\neq l_{2} \end{array}\right. \end{array}\right.$$

Equation (1) can also be written as:

(3) $$\frac{\partial^{2}w}{\partial t^{2}}=\frac{1}{\rho }\frac{\partial \sigma_{N}}{\partial z}+\frac{\delta (t)\delta (z-l_{2})}{\rho }$$

It should be noted that the first expression on the right of Equation (3) represents the acceleration of the sensor generated by internal force, and the second expression represents the acceleration generated by external force. Therefore, we introduce the theoretical density $\rho_{T}$ , $\rho_{T}$ is expressed as follows:

(4) $$\rho_{T}=\rho_{c}V_{c}+\rho_{p}V_{p}$$

Here $\rho_{c}$ , $V_{c}$ and $\rho_{p}$ , $V_{p}$ are, respectively, the density and volume fraction of the cement and piezoelectric material constituting the sensor. Then Equation (3) can be written as:

(5) $$\frac{\partial^{2}w}{\partial t^{2}}=\frac{1}{\rho }\frac{\partial \sigma_{N}}{\partial z}+\frac{\delta (t)\delta (z-l_{2})}{\rho_{T}}$$

For the cement layer C#1 ( $0\leq z\leq l_{1}$ ), according to Equation (5) and without considering the body force and body charge, the basic equations can be written as:

(6) $$\left\{ \begin{array}{l} \frac{\partial^{2}w_{c}}{\partial t^{2}}=\frac{1}{\rho_{c}}\frac{\partial \sigma_{zc}}{\partial z}+\frac{\delta (t)\delta (z-l_{2})}{\rho_{T}}\\ \sigma_{zc}=C_{33c}\varepsilon_{zc}\\ \varepsilon_{zc}=\frac{\partial w_{c}}{\partial z} \end{array}\right.$$

Here $C_{33c}$ , $\rho_{c}$ and $w_{c}$ are, respectively, the elastic stiffness coefficient, density and displacement of the cement material. Equations (2) and (6) are combined to give the following equation:

(7) $$\frac{\partial^{2}w_{c}}{\partial t^{2}}-{C_{a}}^{2}\frac{\partial^{2}w_{c}}{\partial z^{2}}=0$$

Here $C_{a}=\sqrt{C_{33c}∕\rho_{c}}$ represents the propagation velocity of the vibration wave in the cement layer. For the piezoelectric layer P#2 ( $l_{1}\leq z\leq l_{2}$ ), according to Equation (5) and without considering the body force and body charge, the basic equations can also be written as follows:

(8) $$\left\{ \begin{array}{l} \frac{\partial^{2}w_{p}}{\partial t^{2}}=\frac{1}{\rho_{p}}\frac{\partial \sigma_{zp}}{\partial z}+\frac{\delta (t)\delta (z-l_{2})}{\rho_{T}}\\ \sigma_{zp}=C_{33p}\varepsilon_{zp}-e_{33}E_{z}\\ \varepsilon_{zp}=\frac{\partial w_{p}}{\partial z} \end{array}\right.$$

(9) $$\left\{ \begin{array}{l} D_{z}=e_{33}\varepsilon_{zp}+\varepsilon_{33}^{S}E_{z}\\ E_{z}=-\frac{\partial \phi }{\partial z}\\ \frac{\partial D_{z}}{\partial z}=0 \end{array}\right.$$

Here $C_{33p}$ , $e_{33}$ , $\varepsilon_{33}^{S}$ and $w_{p}$ are, respectively, the elastic stiffness coefficient, piezoelectric coefficient, permittivity coefficient and displacement of the piezoelectric material.

Equations (2), (8) and (9) are combined to give the following equations:

(10) $$\left\{ \begin{array}{l} \frac{\partial^{2}w_{p}}{\partial t^{2}}=\frac{C_{33p}}{\rho_{p}}\frac{\partial^{2}w_{p}}{\partial z^{2}}+\frac{e_{33}}{\rho_{p}}\frac{\partial^{2}\phi }{\partial z^{2}}+\frac{\delta (t)\delta (z-l_{2})}{\rho_{T}}\\ \varepsilon_{33}^{S}\frac{\partial^{2}\phi }{\partial z^{2}}=e_{33}\frac{\partial^{2}w_{p}}{\partial z^{2}} \end{array}\right.$$

which could be rewritten as:

(11) $$\frac{\partial^{2}w_{p}}{\partial t^{2}}-{C_{b}}^{2}\frac{\partial^{2}w_{p}}{\partial z^{2}}=\frac{\delta (t)}{\rho_{T}}\delta (z-l_{2})$$

Here $C_{b}=\sqrt{E_{0}∕\rho_{p}}$ represents the propagation velocity of the vibration wave in the piezoelectric layer, here, $E_{0}{=C}_{33p}{{+e}_{33}}^{2}∕\varepsilon_{33}^{S}$ .

Considering the initial conditions and boundary conditions of the sensor, the equation of motion and the definite conditions are summarized as follows:

{(12a)∂2wc∂t2−Ca2∂2wc∂z2=0 ;0≤z≤l1(12b)∂2wp∂t2−Cb2∂2wp∂z2=δ(t)ρTδ(z−l2); l1≤z≤l2(12c)wc(z,0)=∂wc(z,0)∂t=0; 0≤z≤l2(12d)wp(z,0)=∂wp(z,0)∂t=0; 0≤z≤l2(12e)wc(0,t)=∂wp(l2,t)∂z=0; t≥0(12f)wc(l1,t)=wp(l1,t); t≥0(12g)C33c∂wc(l1,t)∂z=E0∂wp(l1,t)∂z; t≥0(12h)D≡0

where $E_{0}$ is the modulus of elasticity of the piezoelectric material.

## 3. Theoretical Solutions of 2-2 Cement-Based Piezoelectric Dual-Layer Stacked Sensor under Impact Load

In this section, the exact solution of a 2-2 cement-based piezoelectric dual-layer stacked sensor can be obtained by utilizing the variable separation method (also known as standing wave method) and the Duhamel integral. Firstly, the displacement of the cement and piezoelectric material can be decomposed as follows:

(13) $$\left\{ \begin{array}{l} w_{c}(z,t)=Z_{c}(z)T_{c}(t)\\ w_{p}(z,t)=Z_{p}(z)T_{p}(t) \end{array}\right.$$

Substituting the above equations into Equation (12), the eigenvalue problem of original definite problem and the frequency equations can be obtained as follows:

{(14a)Zc″(z)+λ1Zc(z)=0; 0≤z≤l1(14b)Zp″(z)+λ2Zp(z)=0; l1≤z≤l2(14c)Zc(l1)=Zp(l1); C33cZc′(l1)=E0Zp′(l1)(14d)Zc(0)=0; Zp′(l2)=0

(15) $$\left\{ \begin{array}{l} {T_{c}}^{\prime \prime }(t)+\lambda_{1}{C_{a}}^{2}T_{c}(t)=0;\;t\geq 0\\ {T_{p}}^{\prime \prime }(t)+\lambda_{2}{C_{b}}^{2}T_{p}(t)=0;\;t\geq 0 \end{array}\right.$$

Combining Equation (12f) with Equation (15) gives the following relation:

(16) $$\sqrt{\lambda_{1}}C_{a}=\sqrt{\lambda_{2}}C_{b}$$

Solving Equations (14a) and (14b) obtains the following solutions:

(17) $$\left\{ \begin{array}{l} Z_{cn}(z)=a_{1n}\cos\sqrt{\lambda_{1n}}z+b_{1n}\sin\sqrt{\lambda_{1n}}z;\;0\leq z\leq l_{1}\\ Z_{pn}(z)=a_{2n}\cos\sqrt{\lambda_{2n}}z+b_{2n}\sin\sqrt{\lambda_{2n}}z;\;l_{1}\leq z\leq l_{2} \end{array}\right.$$

in which $a_{1n}$ , $b_{1n}$ , $a_{2n}$ , $b_{2n}$ are undetermined coefficients. Substitution of Equation (17) into Equations (14c) and (14d) leads to the following equations:

(18) $$\left\{ \begin{array}{l} a_{1n}=0\\ a_{2n}\sin\sqrt{\lambda_{2n}}l_{2}-b_{2n}\cos\sqrt{\lambda_{2n}}l_{2}=0\\ a_{1n}\cos\sqrt{\lambda_{1n}}l_{1}+b_{1n}\sin\sqrt{\lambda_{1n}}l_{1}-a_{2n}\cos\sqrt{\lambda_{2n}}l_{1}-b_{2n}\sin\sqrt{\lambda_{2n}}l_{1}=0\\ C_{33c}\sqrt{\lambda_{1n}}(a_{1n}\sin\sqrt{\lambda_{1n}}l_{1}-b_{1n}\cos\sqrt{\lambda_{1n}}l_{1})-E_{0}\sqrt{\lambda_{2n}}(a_{2n}\sin\sqrt{\lambda_{2n}}l_{1}-b_{2n}\cos\sqrt{\lambda_{2n}}l_{1})=0 \end{array}\right.$$

Solving Equation (18) obtains the following solutions:

(19) $$\left\{ \begin{array}{l} a_{1n}=0\\ b_{1n}=1\\ a_{2n}=\sin\sqrt{\lambda_{1n}}l_{1}\cos\sqrt{\lambda_{2n}}l_{1}-\frac{C_{33c}\sqrt{\lambda_{1n}}}{E_{0}\sqrt{\lambda_{2n}}}\cdot \cos\sqrt{\lambda_{1n}}l_{1}\sin\sqrt{\lambda_{2n}}l_{1}\\ b_{2n}=\sin\sqrt{\lambda_{1n}}l_{1}\sin\sqrt{\lambda_{2n}}l_{1}+\frac{C_{33c}\sqrt{\lambda_{1n}}}{E_{0}\sqrt{\lambda_{2n}}}\cdot \cos\sqrt{\lambda_{1n}}l_{1}\cos\sqrt{\lambda_{2n}}l_{1} \end{array}\right.$$

In order to make $a_{1n}$ , $b_{1n}$ , $a_{2n}$ , $b_{2n}$ have untrivial solutions, let the coefficient determinant of Equation (18) equals to zero:

(20) $$\left|\begin{array}{cccc} 1 & 0 & 0 & 0\\ 0 & 0 & \sin\sqrt{\lambda_{2n}}l_{2} & -\cos\sqrt{\lambda_{2n}}l_{2}\\ \cos\sqrt{\lambda_{1n}}l_{1} & \sin\sqrt{\lambda_{1n}}l_{1} & -\cos\sqrt{\lambda_{2n}}l_{1} & -\sin\sqrt{\lambda_{2n}}l_{1}\\ C_{33c}\sqrt{\lambda_{1n}}\sin\sqrt{\lambda_{1n}}l_{1} & -C_{33c}\sqrt{\lambda_{1n}}\cos\sqrt{\lambda_{1n}}l_{1} & -E_{0}\sqrt{\lambda_{2n}}\sin\sqrt{\lambda_{2n}}l_{1} & E_{0}\sqrt{\lambda_{2n}}\cos\sqrt{\lambda_{2n}}l_{1} \end{array}\right|=0$$

That means that the following formula must be satisfied:

(21) $$C_{33c}\sqrt{\lambda_{1n}}\cos\sqrt{\lambda_{1n}}l_{1}\cos\sqrt{\lambda_{2n}}h_{2}-E_{0}\sqrt{\lambda_{2n}}\sin\sqrt{\lambda_{1n}}l_{1}\sin\sqrt{\lambda_{2n}}h_{2}=0$$

where $h_{2}{=l}_{2}{-l}_{1}$ . On the basis of Equation (16), we can obtained the relation $\sqrt{\lambda_{1n}}C_{a}=\sqrt{\lambda_{2n}}C_{b}=\sqrt{\bar{\lambda_{n}}}$ . Then we define:

(22) $$t_{1}=\frac{l_{1}}{C_{a}},\;t_{2}=\frac{h_{2}}{C_{b}},\;t_{1}+t_{2}=T_{0},\;\bar{t_{1}}=\frac{t_{1}}{T_{0}},\;\bar{t_{2}}=\frac{t_{2}}{T_{0}},\;\sqrt{\bar{\lambda_{n}}}T_{0}=\sqrt{\bar{\bar{\lambda_{n}}}}$$

Utilizing Equation (22) and relation $\sqrt{\lambda_{1n}}C_{a}=\sqrt{\lambda_{2n}}C_{b}=\sqrt{\bar{\lambda_{n}}}$ , we can obtain that $\sqrt{\lambda_{1n}}l_{1}=\sqrt{\bar{\bar{\lambda_{n}}}}\bar{t_{1}}$ , $\sqrt{\lambda_{2n}}h_{2}=\sqrt{\bar{\bar{\lambda_{n}}}}\bar{t_{2}}$ , $\sqrt{\lambda_{1n}}=\sqrt{\lambda_{2n}}\cdot \frac{C_{b}}{C_{a}}$ and $\sqrt{\lambda_{1n}}=\frac{\sqrt{\bar{\bar{\lambda_{n}}}}}{T_{0}C_{a}}$ , $\sqrt{\lambda_{2n}}=\frac{\sqrt{\bar{\bar{\lambda_{n}}}}}{T_{0}C_{b}}$ . Thus Equation (21) can be simplified as the dimensionless characteristic equation:

(23) $$C_{33c}C_{b}\cos\sqrt{\bar{\bar{\lambda_{n}}}}\bar{t_{1}}\cos\sqrt{\bar{\bar{\lambda_{n}}}}\bar{t_{2}}-E_{0}C_{a}\sin\sqrt{\bar{\bar{\lambda_{n}}}}\bar{t_{1}}\sin\sqrt{\bar{\bar{\lambda_{n}}}}\bar{t_{2}}=0$$

After obtaining the value of $\sqrt{\bar{\bar{\lambda_{n}}}}$ by the equation above, $\sqrt{\lambda_{1n}}$ and $\sqrt{\lambda_{2n}}$ can be obtained by $\sqrt{\lambda_{1n}}=\frac{\sqrt{\bar{\bar{\lambda_{n}}}}}{T_{0}C_{a}}$ and $\sqrt{\lambda_{2n}}=\frac{\sqrt{\bar{\bar{\lambda_{n}}}}}{T_{0}C_{b}}$ , respectively, so the corresponding eigenfunctions can be obtained.

According to the variable separation method:

(24) $$\left\{ \begin{array}{l} w_{c}(z,t)=\sum_{n=1}^{\infty }T_{n}(t)\cdot \sin\sqrt{\lambda_{1n}}z\\ w_{p}(z,t)=\sum_{n=1}^{\infty }T_{n}(t)\cdot (a_{2n}\cos\sqrt{\lambda_{2n}}z+b_{2n}\sin\sqrt{\lambda_{2n}}z) \end{array}\right.$$

where n=1,2,3,⋯ . Substituting Equation (24) into Equations (12a) and (12b) leads to the following equations:

{(25a)∑n=1∞[Tn″(t)+λn¯Tn(t)]⋅sinλ1nz=0(25b)∑n=1∞[Tn″(t)+λn¯Tn(t)]⋅(a2ncosλ2nz+b2nsinλ2nz)=δ(t)ρTδ(z−l2)

Using $\sin\sqrt{\lambda_{1m}}z$ to multiplied both sides of Equation (25a), and taken definite integral $\int_{0}^{l_{1}}dz$ ; using $a_{2m}\cos\sqrt{\lambda_{2m}}{z+b}_{2m}\sin\sqrt{\lambda_{2m}}z$ to multiplied both sides of Equation (25b), and taken definite integral $\int_{l_{1}}^{l_{2}}dz$ , then added these two equations. Combining the weighted orthogonality of the eigenfunctions and we can obtained the following equations:

(26) $$\left\{ \begin{array}{l} \;\\ \int_{0}^{l_{1}}(\sin\sqrt{\lambda_{1n}}z\cdot \sin\sqrt{\lambda_{1m}}z)dz=0;\;n\neq m\\ \int_{l_{1}}^{l_{2}}(a_{2n}\cos\sqrt{\lambda_{2n}}z+b_{2n}\sin\sqrt{\lambda_{2n}}z)\cdot (a_{2m}\cos\sqrt{\lambda_{2m}}z+b_{2m}\sin\sqrt{\lambda_{2m}}z)dz=0;\;n\neq m\\ \int_{0}^{l_{1}}{\left(\sin\sqrt{\lambda_{1n}}z\right)}^{2}dz=\frac{l_{1}}{2}-\frac{\sin2\sqrt{\lambda_{1n}}l_{1}}{4\sqrt{\lambda_{1n}}};\;n=m\\ \int_{l_{1}}^{l_{2}}{\left(a_{2n}\cos\sqrt{\lambda_{2n}}z+b_{2n}\sin\sqrt{\lambda_{2n}}z\right)}^{2}dz=a_{2n}b_{2n}\frac{\cos2\sqrt{\lambda_{2n}}l_{1}-\cos2\sqrt{\lambda_{2n}}l_{2}}{2\sqrt{\lambda_{2n}}}+\\ {a_{2n}}^{2}(\frac{h_{2}}{2}+\frac{\sin2\sqrt{\lambda_{2n}}l_{2}-\sin2\sqrt{\lambda_{2n}}l_{1}}{4\sqrt{\lambda_{2n}}})+{b_{2n}}^{2}(\frac{h_{2}}{2}-\frac{\sin2\sqrt{\lambda_{2n}}l_{2}-\sin2\sqrt{\lambda_{2n}}l_{1}}{4\sqrt{\lambda_{2n}}});\;n=m \end{array}\right.$$

(27) $$\int_{l_{1}}^{l_{2}}\frac{\delta (t)}{\rho_{T}}\delta (z-l_{2})\cdot (a_{2m}\cos\sqrt{\lambda_{2m}}z+b_{2m}\sin\sqrt{\lambda_{2m}}z)dz=\left\{ \begin{array}{l} \frac{\delta (t)}{\rho_{T}}\cdot (a_{2n}\cos\sqrt{\lambda_{2n}}l_{2}+b_{2n}\sin\sqrt{\lambda_{2n}}l_{2});\;n=m\\ \frac{\delta (t)}{\rho_{T}}\cdot (a_{2m}\cos\sqrt{\lambda_{2m}}l_{2}+b_{2n}\sin\sqrt{\lambda_{2m}}l_{2});\;n\neq m \end{array}\right.$$

So when n=m , the following equation can be obtained ( n≠m meaningless):

(28) $${T_{n}}^{\prime \prime }(t)+\bar{\lambda_{n}}T_{n}(t)=\frac{\delta (t)}{M_{n}\rho_{T}}\cdot (a_{2n}\cos\sqrt{\lambda_{2n}}l_{2}+b_{2n}\sin\sqrt{\lambda_{2n}}l_{2})$$

where:

(29) $$M_{n}=\int_{0}^{l_{1}}{\left(\sin\sqrt{\lambda_{1n}}z\right)}^{2}dz+\int_{l_{1}}^{l_{2}}{\left(a_{2n}\cos\sqrt{\lambda_{2n}}z+b_{2n}\sin\sqrt{\lambda_{2n}}z\right)}^{2}dz$$

Since the initial conditions of the sensor is zero, the Equation (28) can be solved by the Duhamer integral formula:

(30) $$\begin{array}{l} T_{n}(t)=\frac{\left(a_{2n}\cos\sqrt{\lambda_{2n}}l_{2}+b_{2n}\sin\sqrt{\lambda_{2n}}l_{2}\right)}{M_{n}\rho_{T}\sqrt{\bar{\lambda_{n}}}}\cdot \int_{0}^{t}\delta (\tau )\sin\sqrt{\bar{\lambda_{n}}}(t-\tau )d\tau \\ \;=\frac{\left(a_{2n}\cos\sqrt{\lambda_{2n}}l_{2}+b_{2n}\sin\sqrt{\lambda_{2n}}l_{2}\right)}{M_{n}\rho_{T}\sqrt{\bar{\lambda_{n}}}}\cdot \sin\sqrt{\bar{\lambda_{n}}}t \end{array}$$

Therefore, the exact solutions of the displacement of 2-2 cement-based piezoelectric dual-layer stacked sensor under impact load can be obtained as:

(31) $$\left\{ \begin{array}{l} w_{c}(z,t)=\sum_{n=1}^{\infty }\frac{D_{n}}{\sqrt{\bar{\lambda_{n}}}}\cdot \sin\sqrt{\bar{\lambda_{n}}}t\sin\sqrt{\lambda_{1n}}z;\;t\geq 0,\;0\leq z\leq l_{1}\\ w_{p}(z,t)=\sum_{n=1}^{\infty }\frac{D_{n}}{\sqrt{\bar{\lambda_{n}}}}\cdot \sin\sqrt{\bar{\lambda_{n}}}t(a_{2n}\cos\sqrt{\lambda_{2n}}z+b_{2n}\sin\sqrt{\lambda_{2n}}z);\;t\geq 0,\;l_{1}\leq z\leq l_{2} \end{array}\right.$$

where $D_{n}=\frac{{(a}_{2n}\cos\sqrt{\lambda_{2n}}l_{2}{+b}_{2n}\sin\sqrt{\lambda_{2n}}l_{2})}{M_{n}\rho_{T}}$ ; n=1,2,3,⋯ .

Combining Equations (6), (8), (9) and (12h), the exact precise solutions of the mechanical and electrical quantities of 2-2 cement-based piezoelectric dual-layer stacked sensor under impact load can be obtained as follows:

Stress functions:

(32) $$\left\{ \begin{array}{l} \sigma_{c}(z,t)=\sum_{n=1}^{\infty }\frac{C_{33c}D_{n}\sqrt{\lambda_{1n}}}{\sqrt{\bar{\lambda_{n}}}}\cdot \sin\sqrt{\bar{\lambda_{n}}}t\cos\sqrt{\lambda_{1n}}z;\;t\geq 0,\;0\leq z\leq l_{1}\\ \sigma_{p}(z,t)=\sum_{n=1}^{\infty }\frac{E_{0}D_{n}\sqrt{\lambda_{2n}}}{\sqrt{\bar{\lambda_{n}}}}\cdot \sin\sqrt{\bar{\lambda_{n}}}t(b_{2n}\cos\sqrt{\lambda_{2n}}z-a_{2n}\sin\sqrt{\lambda_{2n}}z);\;t\geq 0,\;l_{1}\leq z\leq l_{2} \end{array}\right.$$

Strain functions:

(33) $$\left\{ \begin{array}{l} \varepsilon_{c}(z,t)=\sum_{n=1}^{\infty }\frac{D_{n}\sqrt{\lambda_{1n}}}{\sqrt{\bar{\lambda_{n}}}}\cdot \sin\sqrt{\bar{\lambda_{n}}}t\cos\sqrt{\lambda_{1n}}z;\;t\geq 0,\;0\leq z\leq l_{1}\\ \varepsilon_{p}(z,t)=\sum_{n=1}^{\infty }\frac{D_{n}\sqrt{\lambda_{2n}}}{\sqrt{\bar{\lambda_{n}}}}\cdot \sin\sqrt{\bar{\lambda_{n}}}t(b_{2n}\cos\sqrt{\lambda_{2n}}z-a_{2n}\sin\sqrt{\lambda_{2n}}z);\;t\geq 0,\;l_{1}\leq z\leq l_{2} \end{array}\right.$$

Velocity functions:

(34) $$\left\{ \begin{array}{l} v_{c}(z,t)=\sum_{n=1}^{\infty }D_{n}\cdot \cos\sqrt{\bar{\lambda_{n}}}t\sin\sqrt{\lambda_{1n}}z;\;t\geq 0,\;0\leq z\leq l_{1}\\ v_{p}(z,t)=\sum_{n=1}^{\infty }D_{n}\cdot \cos\sqrt{\bar{\lambda_{n}}}t(a_{2n}\cos\sqrt{\lambda_{2n}}z+b_{2n}\sin\sqrt{\lambda_{2n}}z);\;t\geq 0,\;l_{1}\leq z\leq l_{2} \end{array}\right.$$

Acceleration functions:

(35) $$\left\{ \begin{array}{l} a_{c}(z,t)=\sum_{n=1}^{\infty }-D_{n}\sqrt{\bar{\lambda_{n}}}\cdot \sin\sqrt{\bar{\lambda_{n}}}t\sin\sqrt{\lambda_{1n}}z;\;t\geq 0,\;0\leq z\leq l_{1}\\ a_{p}(z,t)=\sum_{n=1}^{\infty }-D_{n}\sqrt{\bar{\lambda_{n}}}\cdot \sin\sqrt{\bar{\lambda_{n}}}t(a_{2n}\cos\sqrt{\lambda_{2n}}z+b_{2n}\sin\sqrt{\lambda_{2n}}z);\;t\geq 0,\;l_{1}\leq z\leq l_{2} \end{array}\right.$$

Electric potential of piezoelectric layer:

(36) $$\phi (z,t)=\sum_{n=1}^{\infty }\frac{e_{33}\rho_{p}D_{n}\sqrt{\bar{\lambda_{n}}}}{(C_{33p}\varepsilon_{33}^{S}+{e_{33}}^{2})\lambda_{2n}}\cdot \sin\sqrt{\bar{\lambda_{n}}}t\cdot [a_{2n}(\cos\sqrt{\lambda_{2n}}z-\cos\sqrt{\lambda_{2n}}l_{1})+b_{2n}(\sin\sqrt{\lambda_{2n}}z-\sin\sqrt{\lambda_{2n}}l_{1})];\;t\geq 0,\;l_{1}\leq z\leq l_{2}$$

Electric field intensity of piezoelectric layer:

(37) $$E(z,t)=\sum_{n=1}^{\infty }-\frac{e_{33}\rho_{p}D_{n}\sqrt{\bar{\lambda_{n}}}}{(C_{33p}\varepsilon_{33}^{S}+{e_{33}}^{2})\sqrt{\lambda_{2n}}}\cdot \sin\sqrt{\bar{\lambda_{n}}}t\cdot (b_{2n}\cos\sqrt{\lambda_{2n}}z-a_{2n}\sin\sqrt{\lambda_{2n}}z);\;t\geq 0,\;l_{1}\leq z\leq l_{2}$$

Thus, the precise mechanical and electrical fields of 2-2 cement-based piezoelectric dual-layer stacked sensor under impact load have been fully determined by the variable separation method and Duhamel integral.

## 4. Comparison and Discussion

In this section, a numerical simulation of the 2-2 cement-based piezoelectric dual-layer stacked sensor under impact load is presented and compared with the theoretical solutions obtained in the previous sections and Li’s results [25]. The total thickness of the sensor $l_{2}$ is taken as 0.015 m. It is defined that the cement layer and piezoelectric layer are made of ordinary Portland cement and piezoelectric ceramics, respectively. The main material parameters of piezoelectric ceramics are based on Li’s experiments [26]. The related structural and material parameters take the values summarized in Table 1.

**Figure 2:**
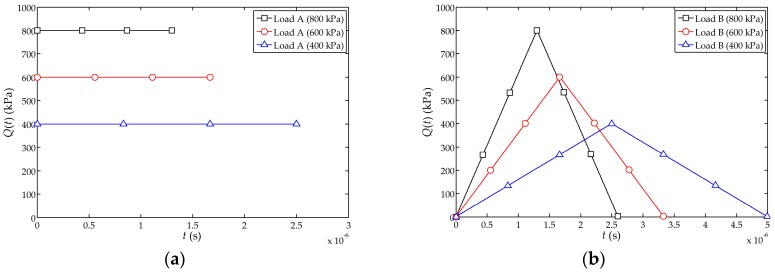

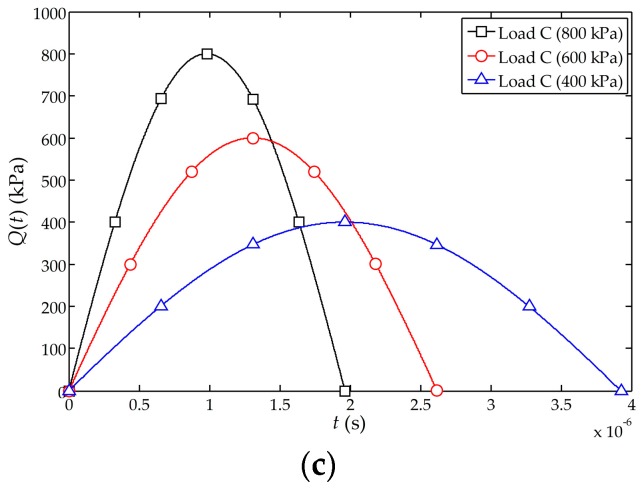
Schematics of the impact loads in the numerical simulation analysis: (**a**) The transient step load; (**b**) The transient isosceles triangle load; (**c**) The transient haversine wave load.

The numerical simulation analysis is modeled by the finite element analysis software, and the size of the model is 0.001 m×0.001 m×0.015 m . The direction of polarization is z -axis. By using the free meshing method, the unit partition of the analysis model is divided into 10, 10 and 300 segments along x , y and z axis, respectively. The upper and lower surfaces of the piezoelectric layer in the z -axis direction are subjected to the piezoelectric coupling. The electric potential of the lower surface of the piezoelectric layer is set to zero. The model is loaded and solved after the symmetrical boundary conditions are set on the four sides of the model. The impact load Q(t) used in this numerical simulation analysis includes three types, namely, the transient step load (denoted as load A), transient isosceles triangle load (denoted as load B) and transient haversine wave load (denoted as load C). The three types of loads are shown in Figure 2 and they all satisfy $\int_{-\infty }^{+\infty }Q(t)dt=1$ .

**Figure 3:**
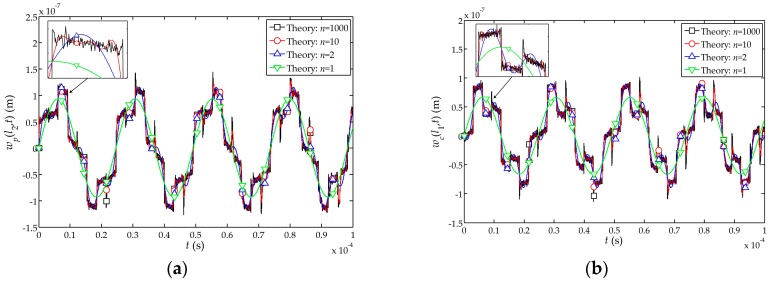
Influences of the impact load δ(t) on displacements of the sensor when n=1 , n=2 , n=10 and n=1000 : (**a**) Influence on displacement $w_{p}{(l}_{2},t)$ at the free end of the sensor; (**b**) Influence on displacement $w_{c}{(l}_{1},t)$ at the interface between the piezoelectric and cement layer.

The theoretical influences of the impact load δ(t) on the displacements $w_{p}{(l}_{2},t)$ and $w_{c}{(l}_{1},t)$ at the free end of the sensor and the interface between the piezoelectric and cement layer when n=1 , n=2 , n=10 and n=1000 are shown in Figure 3. It is noted that the displacement functions of the sensor agrees well for n=1 , 2 , 10 and 1000 . For convenience and without loss of generality, the theoretical solution with n=2 is selected for the following analysis except for special instructions.

**Figure 4:**
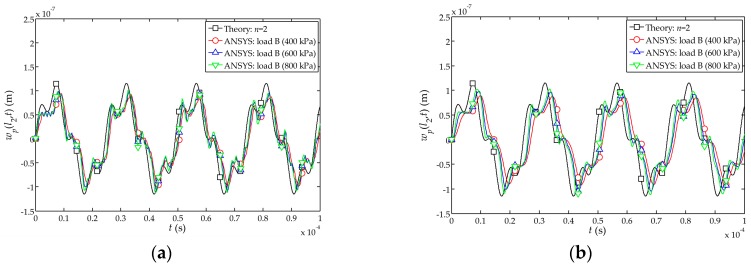

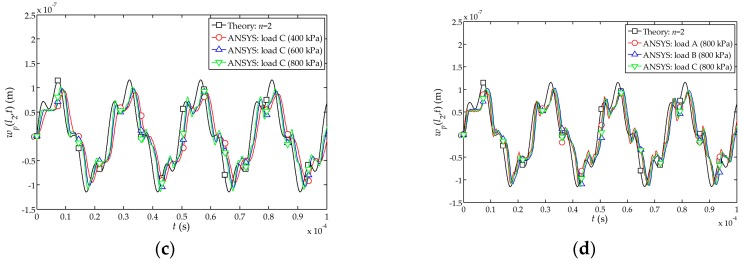
Influences of the impact loads δ(t) and loads A, B, C in theoretical and numerical solutions on the displacement at the free end of the sensor: (**a**) Influence of impact loads δ(t) and load A on $w_{p}{(l}_{2},t)$ ; (**b**) Influence of impact loads δ(t) and load B on $w_{p}{(l}_{2},t)$ ; (**c**) Influence of impact loads δ(t) and load C on $w_{p}{(l}_{2},t)$ ; (**d**) Influence of impact loads δ(t) and loads A, B, C on $w_{p}{(l}_{2},t)$ .

The comparison between the theoretical and numerical solutions of the time-dependent displacement function $w_{p}{(l}_{2},t)$ at the free end of the sensor is shown in Figure 4. It can be found from Figure 4a–c that the numerical simulation and the theoretical solutions are closer when the peak value of the impact loads A, B and C is 800 kPa. Furthermore, when the peak value of impact load is larger, the simulation results become closer to the theoretical solutions.

Figure 4d indicates that the numerical simulation under the load A is slightly better than those obtained under the loads B and C. Therefore, the numerical simulations indicate that the transient step load A (shown in Figure 2a) behaves more closely to the function δ(t) in this theoretical solution.

**Figure 5:**
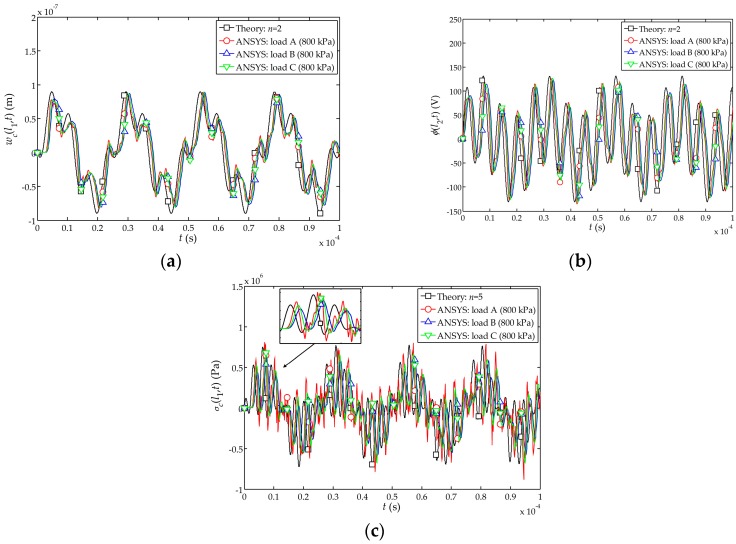
Influences of the impact loads δ(t) and loads A, B, C in theoretical and numerical solutions on the displacement, electric potential and stress of the sensor: (**a**) Influence on displacement $w_{c}{(l}_{1},t)$ at the interface between the piezoelectric and cement layer; (**b**) Influence on electric potential $\phi {(l}_{2},t)$ at the free end; (**c**) Influence on stress $\sigma_{c}{(l}_{1},t)$ at the interface between the piezoelectric and cement layer.

For the sensor under the impact load δ(t) and the load A, B, C with peak value 800 kPa, the displacement $w_{c}{(l}_{1},t)$ , electric potential $\phi {(l}_{2},t)$ and stress $\sigma_{c}{(l}_{1},t)$ are plotted in Figure 5. It can also be seen that the results with the load A are closer to the theoretical solutions as compared to the loads B and C.

**Figure 6:**
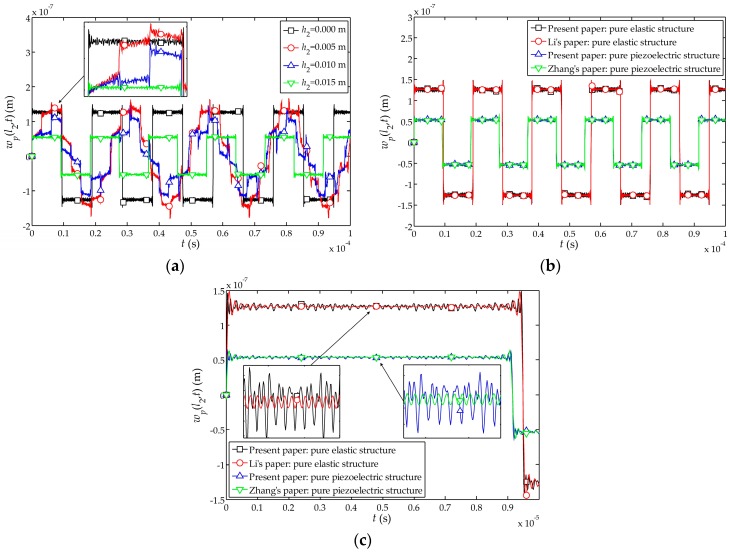
Influences of the thickness of piezoelectric layer $h_{2}$ on the displacement $w_{p}{(l}_{2},t)$ of the sensor: (**a**) Influence on the distribution of the displacement $w_{p}{(l}_{2},t)$ with n=100 ; (**b**) Theoretical comparison with Li’s theory and Zhang’s theory ( n=100 , $0\leq t\leq 1\times 10^{-4}\;s$ ); (**c**) Theoretical comparison with Li’s theory and Zhang’s theory ( n=100 , $0\leq t\leq {1\times 10}^{-5}\;s$ ). The overall thickness of the sensor $l_{2}=0.015\;m$ .

The influences of the thickness of piezoelectric layer on the dynamic characteristics of the sensor are analyzed as follows: the amplitude and period of the displacement of the free end are different for different assumed thicknesses of the piezoelectric layer, namely $h_{2}=0.000\;m$ (pure cement structure), 0.005 m , 0.010 m and 0.015 m (pure piezoelectric structure), as shown in Figure 6a. To keep the overall thickness of the sensor unchanged, the thickness of the cement layer $l_{1}$ is taken as 0.015 m, 0.010 m , 0.005 m and 0 m , respectively. It can be seen that the displacement amplitude and period of the free end are both larger than that of the pure piezoelectric structures, and the thinner the piezoelectric layer, the larger the displacement amplitude and period. Furthermore, Li et al. have obtained the displacement function of the elastic rod under the impact load ([25], pp. 70–74). Zhang et al. have also researched the dynamic characteristics of the pure piezoelectric structure under the impact loading [24]. Comparing the theory at present paper with their theories, as shown in Figure 6b,c, and it can be found that the displacement of the free end of pure cement structure and pure piezoelectric structure are in good agreement with Li’s and Zhang’s theories, respectively. It also shows the rationality of using the theoretical density in Section 2, and the correctness of the theory presented in this paper.

**Figure 7:**
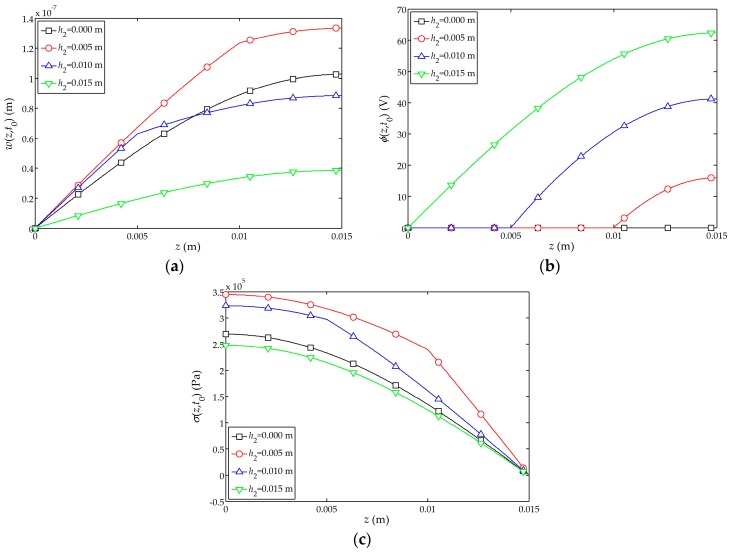
Influences of the thickness of piezoelectric layer $h_{2}$ on the dynamic characteristics of the sensor: (**a**) Influence on the distribution of the displacement ${w(z,t}_{0})$ with n=1 ; (**b**) Influence on the distribution of the electric potential $\phi {(z,t}_{0})$ with n=1 ; (**c**) Influence on the distribution of the stress ${\sigma (z,t}_{0})$ with n=1 .

The distributions of the displacement ${w(z,t}_{0})$ , electric potential $\phi {(z,t}_{0})$ and stress ${\sigma (z,t}_{0})$ along the *z*-axis are shown in Figure 7a–c, respectively. The displacement at the free end of the sensor firstly peaks at $t_{0}$ and $t_{0}=0{.74\times 10}^{-5}\;s$ . It can be found that with the increases of $h_{2}$ , the displacement ${w(z,t}_{0})$ and stress ${\sigma (z,t}_{0})$ of the composite structure and pure piezoelectric structure decreases, and the electric potential of the piezoelectric layer increases as $h_{2}$ increases. When the cement layer is thicker, the overall displacement change is larger; for a pure piezoelectric structure ( $l_{1}=0\;m$ ), the overall displacement of the structure is minimized. This shows that the actuating capability of the cement-based piezoelectric dual-layer stacked sensor is better than that of a pure piezoelectric structure.

**Figure 8:**
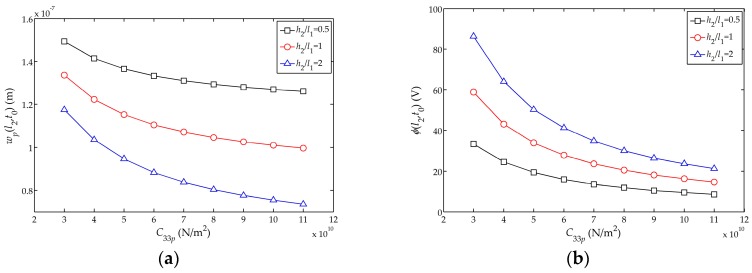

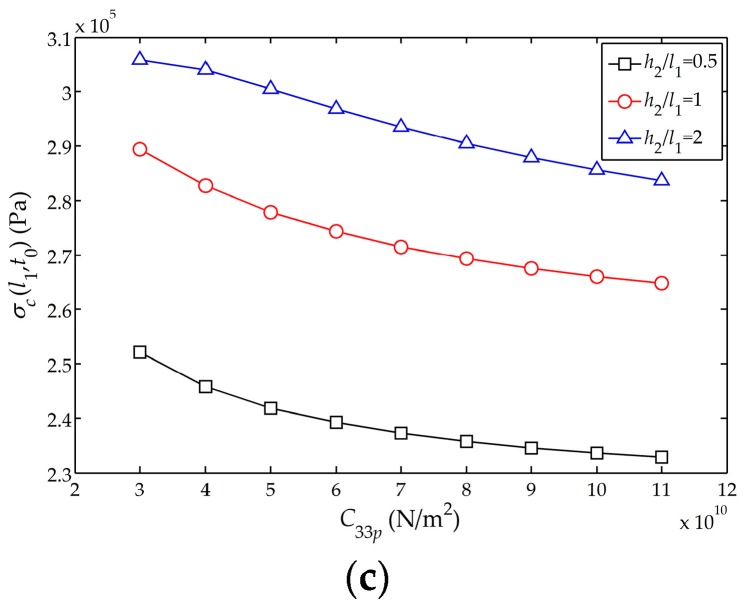
Influences of $C_{33p}$ on the displacement, electric potential and stress amplitudes with the different thickness of piezoelectric layer $h_{2}$ : (**a**) Influence on the displacement amplitude $w_{p}{(l}_{2}{,t}_{0})$ ; (**b**) Influence on the electric potential amplitude $\phi {(l}_{2}{,t}_{0})$ ; (**c**) Influence on the stress amplitude $\sigma_{c}{(l}_{1}{,t}_{0})$ .

The influences of the elastic stiffness $C_{33p}$ on the displacement amplitude $w_{p}{(l}_{2}{,t}_{0})$ , electric potential amplitude $\phi {(l}_{2}{,t}_{0})$ and stress amplitude $\sigma_{c}{(l}_{1}{,t}_{0})$ of the sensor are shown in Figure 8a−c, respectively. It can be found that $w_{p}{(l}_{2}{,t}_{0})$ , $\phi {(l}_{2}{,t}_{0})$ and $\sigma_{c}{(l}_{1}{,t}_{0})$ decrease as the elastic stiffness increases. In addition, with the increasing elastic stiffness, the changing rates of the displacement and electric potential amplitudes of the free end tend to decrease. As for the thicker piezoelectric layer, the rates of change of the displacement amplitude and electric potential amplitude are larger.

**Figure 9:**
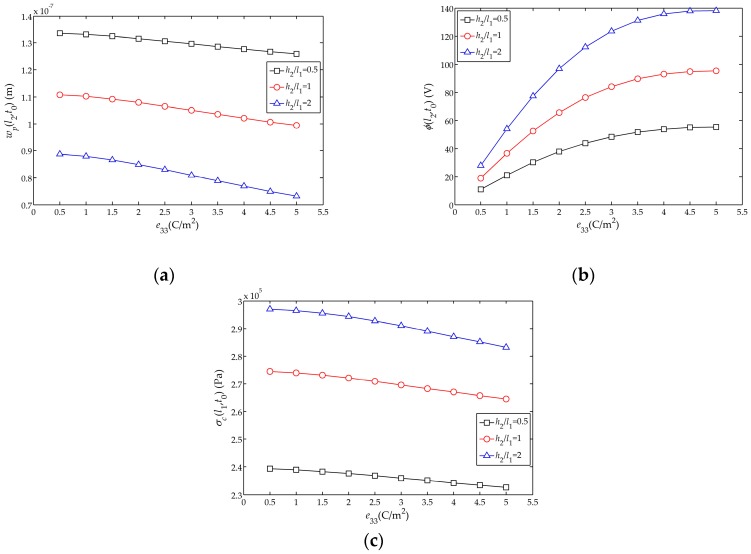
Influences of $e_{33}$ on the displacement, electric potential and stress amplitudes with the different thickness of piezoelectric layer $h_{2}$ : (**a**) Influence on the displacement amplitude $w_{p}{(l}_{2}{,t}_{0})$ ; (**b**) Influence on the electric potential amplitude $\phi {(l}_{2}{,t}_{0})$ ; (**c**) Influence on the stress amplitude $\sigma_{c}{(l}_{1}{,t}_{0})$ .

The influences of the piezoelectric stress constant $e_{33}$ on the displacement amplitude $w_{p}{(l}_{2}{,t}_{0})$ , electric potential amplitude $\phi {(l}_{2}{,t}_{0})$ and stress amplitude $\sigma_{c}{(l}_{1}{,t}_{0})$ of the sensor are shown in Figure 9a–c, respectively. It can be found that with the increases of $e_{33}$ , $w_{p}{(l}_{2}{,t}_{0})$ decreases, with the change smaller for thinner piezoelectric layer. This could be explained as below. From the expression of $C_{b}$ the numerical change of $e_{33}$ has no effect on $C_{b}$ and the influence on the displacement solution in Equation (31) is also small. Besides, with the increasing $e_{33}$ , the electric potential amplitude $\phi {(l}_{2}{,t}_{0})$ of the free end tends to flatten; meanwhile, the variation of stress amplitude $\sigma_{c}{(l}_{1}{,t}_{0})$ is approximately linear with the piezoelectric stress constant $e_{33}$ . Moreover, Figure 9b shows that $e_{33}$ has a great influence on the amplitude of $\phi {(l}_{2}{,t}_{0})$ of the sensor, which ensures that the sensor can produce large electric potential. Like the cases in Figure 8, for a thicker piezoelectric layer, the influence of $e_{33}$ on $\phi {(l}_{2}{,t}_{0})$ , $w_{p}{(l}_{2}{,t}_{0})$ and $\sigma_{c}{(l}_{1}{,t}_{0})$ is larger.

**Figure 10:**
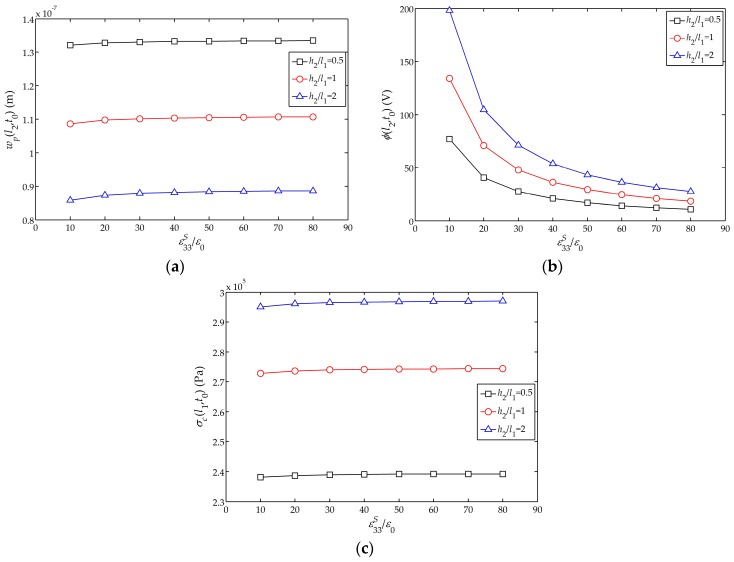
Influences of $\varepsilon_{33}^{S}{/\varepsilon }_{0}$ on the displacement, electric potential and stress amplitudes with the different thickness of piezoelectric layer $h_{2}$ : (**a**) Influence on the displacement amplitude $w_{p}{(l}_{2}{,t}_{0})$ ; (**b**) Influence on the electric potential amplitude $\phi {(l}_{2}{,t}_{0})$ ; (**c**) Influence on the stress amplitude $\sigma_{c}{(l}_{1}{,t}_{0})$ .

Figure 10a–c show the influences of the relative dielectric constant $\varepsilon_{33}^{S}{/\varepsilon }_{0}$ on the displacement amplitude $w_{p}{(l}_{2}{,t}_{1})$ , electric potential amplitude $\phi {(l}_{2}{,t}_{1})$ and stress amplitude $\sigma_{c}{(l}_{1}{,t}_{0})$ of the sensor, respectively. It can be found that with the increase of $\varepsilon_{33}^{S}{/\varepsilon }_{0}$ , $w_{p}{(l}_{2}{,t}_{1})$ increases, though the change is quite small; meanwhile, $\phi {(l}_{2}{,t}_{1})$ decreases rapidly at the beginning and then tends to flatten, and the influence on $\sigma_{c}{(l}_{1}{,t}_{0})$ is negligible. Furthermore, the influence of $\varepsilon_{33}^{S}{/\varepsilon }_{0}$ on $\phi {(l}_{2}{,t}_{1})$ is larger for a thicker piezoelectric layer.

Figure 6, Figure 7, Figure 8, Figure 9 and Figure 10 show the effects of piezoelectric layer thickness and material parameters on the electrical and mechanical behaviors of the sensor in Figure 1a. These results are quite helpful for the design and optimization ofthe 2-2 cement-based piezoelectric dual-layer stacked sensors.

## 5. Conclusions

An analytical study of a 2-2 cement-based piezoelectric dual-layer stacked sensor under impact load is presented based upon the theory of piezoelasticity. Theoretical solutions are obtained by combining all the equations and boundary conditions and utilizing the variable separation method and Duhamel integral. It is found that:

1. The compliance of numerical simulations under the transient step load is slightly better than that under transient isosceles triangle load and transient haversine wave load. The theoretical results show overall good agreement with the numerical results. The numerical simulation are closest to the theoretical results for the larger peak value of the impact load;
2. The displacement amplitude and period of the free end are both larger than that of the pure piezoelectric structures, and the thinner the piezoelectric layer, the larger the displacement amplitude and period;
3. The displacement, electric potential and stress of the considered sensor could be changed/optimized by adjusting the thickness of the piezoelectric layer $h_{2}$ , the elastic stiffness $C_{33p}$ , the piezoelectric stress constant $e_{33}$ and the relative dielectric constant $\varepsilon_{33}^{S}{/\varepsilon }_{0}$ , but the direction, rate and magnitude of the changes are different;
4. By selecting different piezoelectric layer thickness and materials, we could obtain the sensors with desired electrical and mechanical characteristics, which is quite helpful for the design of the cement-based piezoelectric dual-layer stacked sensors.

## Figures and Tables

**Table 1:** The related structural and material parameters of the sensor.

Material	Thickness	Density	Elastic Stiffness Coefficient	Poisson’s Ratio	Piezoelectric Coefficient	Permittivity Coefficient
Ordinary Portland Cement	0.005 m	${2500\;\mathrm{kg}/m}^{3}$	$2{.5\times 10}^{10}\;\mathrm{Pa}$	0.2	/	/
Piezoelectric Ceramics	0.010 m	${5700\;\mathrm{kg}/m}^{3}$	$6{.0\times 10}^{10}\;\mathrm{Pa}$	/	$0{.75\;C/m}^{2}$	$52{.5\;\varepsilon }_{0}$ ^1^

^1^ $\varepsilon_{0}=8{.85\times 10}^{-12}\;F/m$ is the vacuum dielectric constant.
